# Warming reduces the cover and diversity of biocrust-forming mosses and lichens, and increases the physiological stress of soil microbial communities in a semi-arid *Pinus halepensis* plantation

**DOI:** 10.3389/fmicb.2015.00865

**Published:** 2015-08-25

**Authors:** Fernando T. Maestre, Cristina Escolar, Richard D. Bardgett, Jennifer A. J. Dungait, Beatriz Gozalo, Victoria Ochoa

**Affiliations:** ^1^Área de Biodiversidad y Conservación, Departamento de Biología y Geología, Física y Química Inorgánica, Escuela Superior de Ciencias Experimentales y Tecnología, Universidad Rey Juan CarlosMóstoles, Spain; ^2^Faculty of Life Sciences, The University of ManchesterManchester, UK; ^3^Sustainable Soils and Grassland Systems Department, Rothamsted Research, North WykeOkehampton, UK

**Keywords:** biocrusts, climate change, lichen, moss, PLFA, drylands, microbial communities

## Abstract

Soil communities dominated by lichens and mosses (biocrusts) play key roles in maintaining ecosystem structure and functioning in drylands worldwide. However, few studies have explicitly evaluated how climate change-induced impacts on biocrusts affect associated soil microbial communities. We report results from a field experiment conducted in a semiarid *Pinus halepensis* plantation, where we setup an experiment with two factors: cover of biocrusts (low [<15%] *versus* high [>50%]), and warming (control *versus* a ∼2°C temperature increase). Warming reduced the richness and cover (∼45%) of high biocrust cover areas 53 months after the onset of the experiment. This treatment did not change the ratios between the major microbial groups, as measured by phospholipid fatty acid analysis. Warming increased the physiological stress of the Gram negative bacterial community, as indicated by the cy17:0/16:1ω7 ratio. This response was modulated by the initial biocrust cover, as the increase in this ratio with warming was higher in areas with low cover. Our findings suggest that biocrusts can slow down the negative effects of warming on the physiological status of the Gram negative bacterial community. However, as warming will likely reduce the cover and diversity of biocrusts, these positive effects will be reduced under climate change.

## Introduction

Climate change is fostering major shifts in the composition and diversity of biota in terrestrial ecosystems worldwide (Visser and Both, 2005; Rosenzweig et al., 2007; Peñuelas et al., 2013; Buitenwerf et al., 2015). However, large uncertainties exist about how climate change-induced alterations in the composition and diversity of biotic communities will directly impact ecosystem functioning (Hartley et al., 2012; Zhou et al., 2012; Maestre et al., 2013; Delgado-Baquerizo et al., 2014). This is particularly true for terrestrial microbial communities in arid, semi-arid and dry-subhumid environments (drylands), as we are only starting to understand the role that environmental factors such as climate play in determining their abundance, distribution and diversity (e.g., Fierer and Jackson, 2006; Garcia-Pichel et al., 2013; Pasternak et al., 2013; Serna-Chavez et al., 2013). Drylands harbor highly diverse and unique soil microbial communities (Housman et al., 2007; Bates et al., 2010; Steven et al., 2013; Ramirez et al., 2014), which drive ecosystem processes essential for the provision of ecosystem services in these areas, such as nutrient cycling and carbon sequestration (Barrios, 2007; Brussaard, 2012). Understanding how the structure and composition of soil microbial communities will respond to climate change is thus crucial to comprehend the ecological consequences of such change for drylands (Makhalanyane et al., 2015).

Communities composed of eukaryotic algae, cyanobacteria, mosses, liverworts, fungi and lichens (biocrusts) live in the uppermost soil surface in drylands worldwide, where they constitute up to 70% of the living cover (Belnap, 2003). Biocrust constituents exert a strong influence on soil microbial communities beneath them, such as fungi (Bates et al., 2010) and bacteria (Yeager et al., 2004; Castillo-Monroy et al., 2011a), which regulate multiple ecosystem processes (Fitter et al., 2005). Biocrusts fix substantial amounts of atmospheric CO_2_ (>2.6 Pg C/year globally; Elbert et al., 2012), regulate the temporal dynamics of soil CO_2_ eﬄux and net CO_2_ uptake (Wilske et al., 2008; Castillo-Monroy et al., 2011b), affect the activity of soil enzymes involved in C and N cycling (Bowker et al., 2011; Miralles et al., 2013), and regulate other N cycle processes with clear implications for global biogeochemical cycles, such as N fixation (Elbert et al., 2012), nitrification (Castillo-Monroy et al., 2010), and denitrification (Barger et al., 2013). Recent studies suggest that ongoing global warming will negatively impact the photosynthetic activity of soil lichens (Maphangwa et al., 2012) and mosses (Grote et al., 2010), ultimately reducing their growth and dominance within biocrusts (Escolar et al., 2012; Reed et al., 2012; Maestre et al., 2013). Reductions in the abundance of other biocrust-associated cyanobacteria with changes in rainfall patterns have also been reported (Johnson et al., 2012).

While the value of biocrusts for maintaining ecosystem structure and functioning in drylands worldwide is widely recognized (Eldridge and Greene, 1994; Belnap and Lange, 2001; Maestre et al., 2011), the impacts of climate change on biocrust-associated microbial communities have only recently started to receive attention (Johnson et al., 2012; Reed et al., 2012; Yeager et al., 2012; Zelikova et al., 2012; Garcia-Pichel et al., 2013; Maestre et al., 2013; Delgado-Baquerizo et al., 2014). Here we report results from a 53-months field experiment conducted in a semiarid *Pinus halepensis* plantation in south east Spain, where we increased temperature ∼2°C using open top chambers (OTCs) in areas with and without a well-developed biocrust community dominated by lichens. We assessed the effects of warming on the abundance and richness of biocrust-forming mosses and lichens, and tested how these organisms modulated the responses to warming of surface soil (0–1 cm) microbial communities. We hypothesized that warming reduces the cover and diversity of biocrust-forming mosses and lichens (Escolar et al., 2012), and this reduction will increase the impacts of warming on major microbial groups in the soil immediately beneath the crust (Concostrina-Zubiri et al., 2013; Delgado-Baquerizo et al., 2014).

## Materials and Methods

### Study Site

This study was carried out in a *Pinus halepensis* plantation located in the surroundings of Sax, in south east Spain (38° 32′ 15″ N, 0° 49′ 5″ W, 550 m a.s.l.). The climate is Mediterranean semiarid, with average annual temperature and precipitation of 14.6°C and 315 mm respectively (Maestre, 2000). The soil is derived from gypsum, has pH values ∼7, and is classified as a Gypsiric Leptosol (IUSS Working Group WRB, 2006). The vegetation is dominated by *P. halepensis*, which was planted in the 1950s, and also contains grasses and shrubs such as *Stipa tenacissima*, *Anthyllis cytisoides*, and *Helianthemum squamatum.* The open spaces between plants are colonized by a well-developed biocrust community dominated by lichens such as *Diploschistes diacapsis*, *Squamarina lentigera*, *S. cartilaginea*, *Fulgensia subbracteata*, *Toninia sedifolia*, and *Psora decipiens*, and by mosses such as *Tortula revolvens* var. *obtusata* (Maestre et al., 2005).

### Experimental Design

In 15 February 2009, we setup an experiment with two factors and two levels each: biocrust cover (areas with cover of visible biocrust components [mosses and lichens] <15% *versus* areas with cover of mosses and lichens >50%; hereafter low [LC] and high [HC] biocrust cover plots, respectively, Supplementary Figures S1A,B), and warming (control *versus* a ∼2°C annual temperature increase). Ten replicates per combination of treatments were set up, resulting in a total of 40 experimental plots. Field plots were established allowing a buffer distance of 1 m to minimize the risk of sampling no independent areas (Supplementary Figure S1C). The warming treatment aimed to simulate the average of predictions derived from six Atmosphere-Ocean General Circulation Models for the second half of the 21st century (2040–2070) in central and south–eastern Spain (De Castro et al., 2005). For this, we built OTCs using a hexagonal design with the following dimensions (Supplementary Figure S1D): 40 cm × 50 cm × 32 cm. The OTCs were built using methacrylate sheets, which transmit ∼92% of visible light, reflect 4% of incoming radiation and pass on ∼85% of incoming energy (information provided by the manufacturer; Decorplax S. L., Humanes, Spain). These chambers are open on the top to allow entrance of rainfall and air, and are located 5 cm above the surface to allow air flow and avoid excessive temperatures within the chamber (Supplementary Figure S1D). The design of the OTCs is similar to that employed in warming experiments carried out in arctic (Arft et al., 1999) and dryland (Maphangwa et al., 2012) areas, and we have successfully used them in previous studies conducted with lichen-dominated biocrusts (Maestre et al., 2013; Ladrón de Guevara et al., 2014). Air and soil temperatures, and soil moisture were continuously monitored inside and outside the OTCs using automated sensors (HOBO U23 Pro v2 Temp/RH and TMC20-HD sensors, Onset Corp., Pocasset, MA, USA, and EC-5 soil moisture sensors, Decagon Devices Inc., Pullman, WA, USA respectively).

### Biocrust Monitoring and Microbial Analyses

Within each plot, we inserted a PVC collar 5 cm into the soil (20 cm diameter, 8 cm height) for monitoring temporal changes in the total cover and richness of the visible components of the biocrust community (mosses and lichens, Supplementary Figure S1A). The number of moss and lichen species in each collar was recorded *in situ* at the beginning of the experiment and 16 and 53 months after. We also took high resolution photographs during these surveys to estimate total biocrust cover. From these photographs, we estimated the proportion of each PVC collar covered by lichens and mosses by mapping their area with the software GIMP^1^ and ImageJ^2^. Cover estimates obtained with these photographs correlate well with those gathered directly in the field (Maestre et al., 2013).

We collected soil samples (0–1 cm depth), at the beginning of the experiment in all the plots, and 16 and 53 months after in five randomly selected plots per combination of treatments. A composite sample per plot was obtained from four soil samples separated at least 10 cm; these samples were collected outside the PVC collars to avoid perturbing the biocrust community there. We carefully removed visible biocrust components from the soil samples and sieved them (2 mm mesh). After that, samples were immediately frozen at -80°C until phospholipid fatty acid (PLFA) analyses (Frostegård et al., 1991). This technique is useful to evaluate how environmental factors, such as temperature, affect the composition of soil microbial communities (Ramsey et al., 2006; Frostegård et al., 2011), and has been widely used in Mediterranean dryland areas (e.g., Steinberger et al., 1999; Zaady et al., 2010; Ben-David et al., 2011; Bárcenas-Moreno et al., 2014). For these analyses, subsamples of 1.5 g of soil were used. A total of 23 individual PLFAs (*i*14:0, 14:0, *i*15:0, *a*15:0, 15:0, 16:0, 16:1ω7, 10Me16:0, *i*17:0, *a*17:0, *i*17:1ω6; nMe17:0, 17:0, cy17:0, 10Me17:0, 14:0 3OH, 18:0, 18:1, 18:1ω9t, 18:1ω9c, 18:3, cy19:0, 20:0) were extracted and quantified according to Bardgett et al. (1996). We used *i*14:0, *i*15:0, *a*15:0, 10Me16:0, *i*17:0, *a*17:0, 10Me17:0 and 10Me18:0 to represent Gram positive bacteria (Zogg et al., 1997; Zelles, 1999); cy17:0, cy19:0 and 16:1ω7 to represent Gram negative bacteria (Ratledge and Wilkinson, 1988; Frostegård and Bååth, 1996); 18:2ω6 and 16:1ω7 as indicators of fungal and cyanobacterial biomass, respectively (Federle, 1986; Bodelier et al., 2009; Mortillaro et al., 2012); and 10Me16:0, 10Me17:0 and 10Me18:0 to represent actinobacteria (White et al., 1997). *i*14:0, *i*15:0, *a*15:0, 10Me16:0, *i*17:0, *a*17:0, 10Me17:0, 10Me18:0, 16:1ω7, 18:1ω7, cy17:0 and cy19:0 represented total bacterial PLFA (Frostegård et al., 1993). The ratio of 18:2ω6: total bacterial PLFAs represented the ratio of fungal: bacterial biomass (Frostegård et al., 1993; Bardgett et al., 1996). The cy17:0/16:1ω7 ratio was used as an indicator of the physiological stress status of microbial communities (Guckert et al., 1986; Kaur et al., 2005; Ben-David et al., 2011).

### Statistical Analyses

To assess the changes in biocrust cover and richness through time, we estimated a difference index (Dif) as *R*_final_ – *R*_initial_, where *R* is the value of the variable of interest in 18 July 2013 (final) and 15 February 2009 (initial). Changes in these variables between these surveys, as measured with Dif, followed a normal distribution but did not show homogeneity of variances. Thus, we evaluated the effects of warming (WA) and biocrust cover (CO), and their interaction, on Dif data using the semi-parametric permutational multivariate analysis of variance (PERMANOVA, Anderson, 2001). This method is based on the use of permutation tests to obtain *p* values, does not rely on the normality assumption of ANOVA, and can handle experimental designs such as those used here. If we assume that the sampling units (experimental plots) are exchangeable among the different treatments, the null hypothesis tested by PERMANOVA is H0: “the centroids of the groups, as defined in the space of the chosen resemblance measure, are equivalent for all treatments” (Anderson and Walsh, 2013). Thus, if this null hypothesis holds true, any observed differences among the centroids in a given dataset will be comparable in size to what would be obtained under random allocation of individual experimental plots to the different treatments (i.e., under permutation; Anderson and Walsh, 2013). For these analyses, the Euclidean distance and 10,000 permutations (permutation of raw data, Anderson and Ter Braak, 2003) were used to analyze our data. Both WA and CO were considered as fixed factors in PERMANOVA analyses. In addition, we evaluated whether median Dif values obtained for each treatment and variable were different from zero using the non-parametric Wilcoxon signed-rank test.

Microbial community composition data (PLFA matrix containing the 23 individual fatty acids measured) obtained at the beginning of the experiment and 16 and 53 months after were analyzed using the PERMANOVA model described above, but based on the Bray–Curtis distance. To aid in the interpretation of these analyses, we created a non-metric multi-dimensional scaling (NMDS) ordination of the PLFA data using this distance. Analyses of the whole PLFA matrix were followed up by analyses of the major microbial groups (Gram positive bacteria, Gram negative bacteria, fungi, actinobacteria, total bacteria, cyanobacteria and the fungi: bacteria ratio) and of the cy17:0/16:1ω7 ratio. These were done separately for each sampling period by using a two-way (WA and CO) ANOVA, with both factors being fixed.

Permutational multivariate analysis of variance analyses were carried out with the PERMANOVA+ for PRIMER statistical package (PRIMER-E Ltd., Plymounth Marine Laboratory, UK). NMDS analyses were performed using the PRIMER package. ANOVA and ANCOVA analyses were carried out using SPSS v. 15.0 statistical software (SPSS Inc., Chicago, IL, USA). Raw biocrust cover and PLFA data are available from figshare (Maestre et al., 2015).

## Results

Throughout the experiment, the warming treatment increased average air and soil temperature by 1.9°C and 1.3°C, respectively (Supplementary Figure S2). Warming effects were maximized during summer (June–September), where air temperatures where increased by warming up to 5°C in some days (Supplementary Figure S2). On average, warming reduced surface soil moisture by 1.3% (Supplementary Figure S3), and the length of periods with relative air humidity of 100% by 44% (Supplementary Figure S4).

### Changes in Biocrust Cover and Richness

**FIGURE 1:**
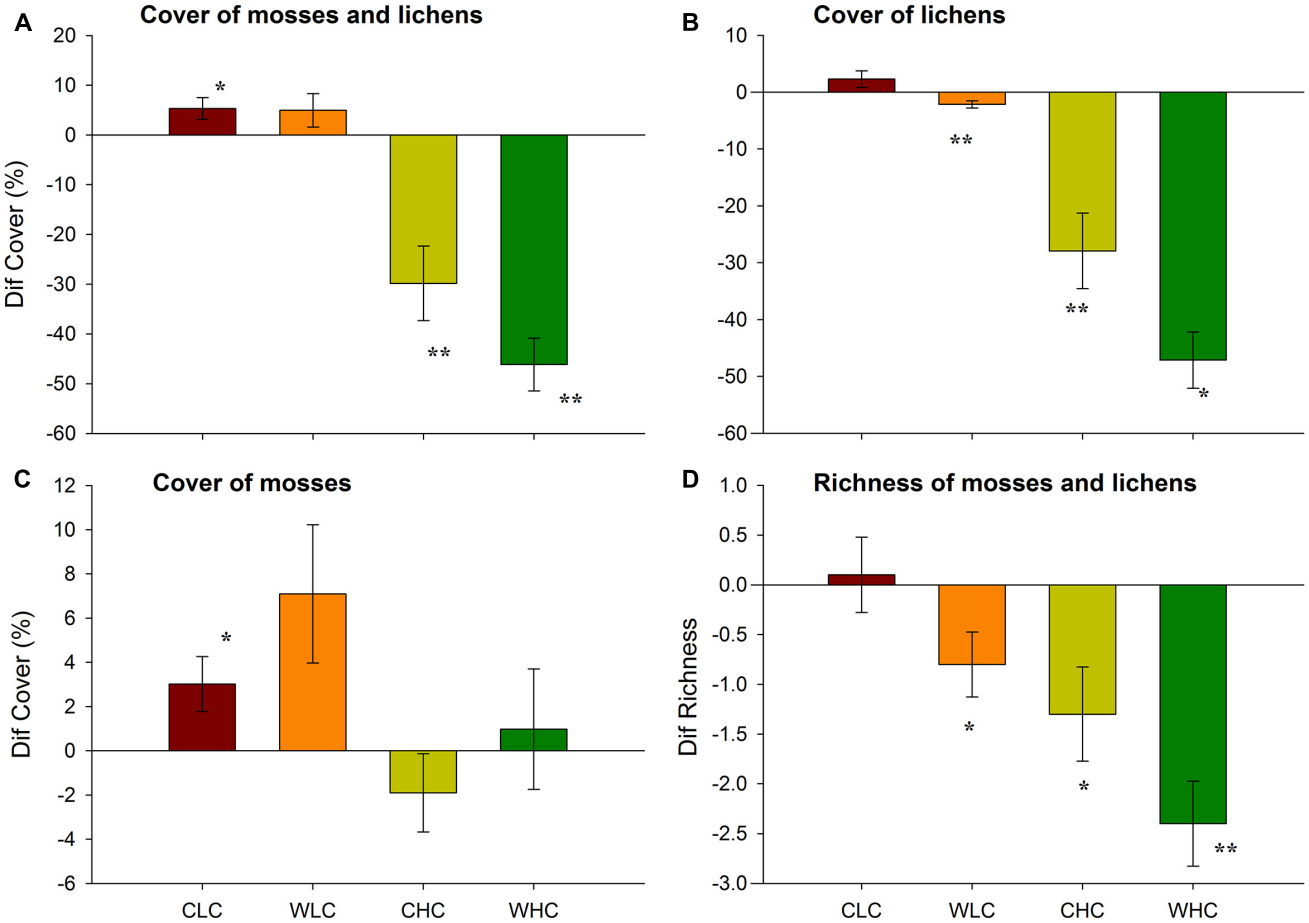
**Differences (Dif) in the total cover of the whole biocrust community (mosses + lichens, A), lichens (B) and mosses (C), and in biocrust richness (D) in areas with initial low and high biocrust cover between February 2009 and June 2013.** Data represent means + SE (*n* = 10). CLC, control low biocrust cover, WLC, warming low biocrust cover, CHC, control high biocrust cover, WHC, warming high biocrust cover. ^∗^ indicate results deviating from 0 (non-parametric Wilcoxon signed-rank test; ^∗^*P* < 0.05, ^∗∗^*P* < 0.01).

At the end of our experiment, the biocrust cover in the LC and HC plots was 10.5/4.9 and 52.1/31.1% for the control and warming treatments, respectively. Across all treatments, we observed a 5% increase and 37% decrease of biocrust cover in LC and HC plots, respectively, at the end of our experiment (**Figure 1A**; PERMANOVA, pseudo-*F*_CO_ = 74.04, *P* < 0.001). This response was not modified by WA (pseudo-*F*_WA_ = 2.78, *P* = 0.102; pseudo-*F*_CO__×__WA_ = 2.54, *P* = 0.126). The increase in moss and lichen cover observed in the LC control plots, and the decrease observed in the HC plots, was significant (**Figure 1A**). The analysis of the changes in cover for lichens alone yielded similar results to those described for the whole biocrust community, albeit a significant decrease in lichen cover was also observed with WA in the LC plots (**Figure 1B**; PERMANOVA, pseudo-*F*_CO_ = 79.08, *P* < 0.001). In this case, plots subjected to warming showed a significant decrease in lichen cover as compared to control plots (PERMANOVA, pseudo-*F*_WA_ = 7.83, *P* = 0.006). The analysis of variations in the cover of mosses showed a different picture, as these were not affected by WA (**Figure 1C**; PERMANOVA, pseudo-*F*_WA_ = 2.22, *P* = 0.144; pseudo-*F*_CO__×__WA_ = 0.07, *P* = 0.790). However, this cover increased in LC, but not in HC, plots (PERMANOVA, pseudo-*F*_CO_ = 5.57, *P* = 0.019).

At the beginning of the experiment, a total of 12 species of lichens and mosses were identified (Supplementary Table S1). After 53 months, biocrust species richness significantly decreased with WA, regardless of the initial biocrust cover (**Figure 1D**; PERMANOVA, pseudo-*F*_WA_ = 6.10, *P* = 0.016; pseudo-*F*_CO__×__WA_ = 0.06, *P* = 0.847). Significant differences were observed between LC and HC plots (PERMANOVA, pseudo-*F*_CO_ = 13.73, *P* = 0.009), with declines in biocrust richness observed particularly in the latter.

### Changes in the Microbial PLFA Composition

**FIGURE 2:**
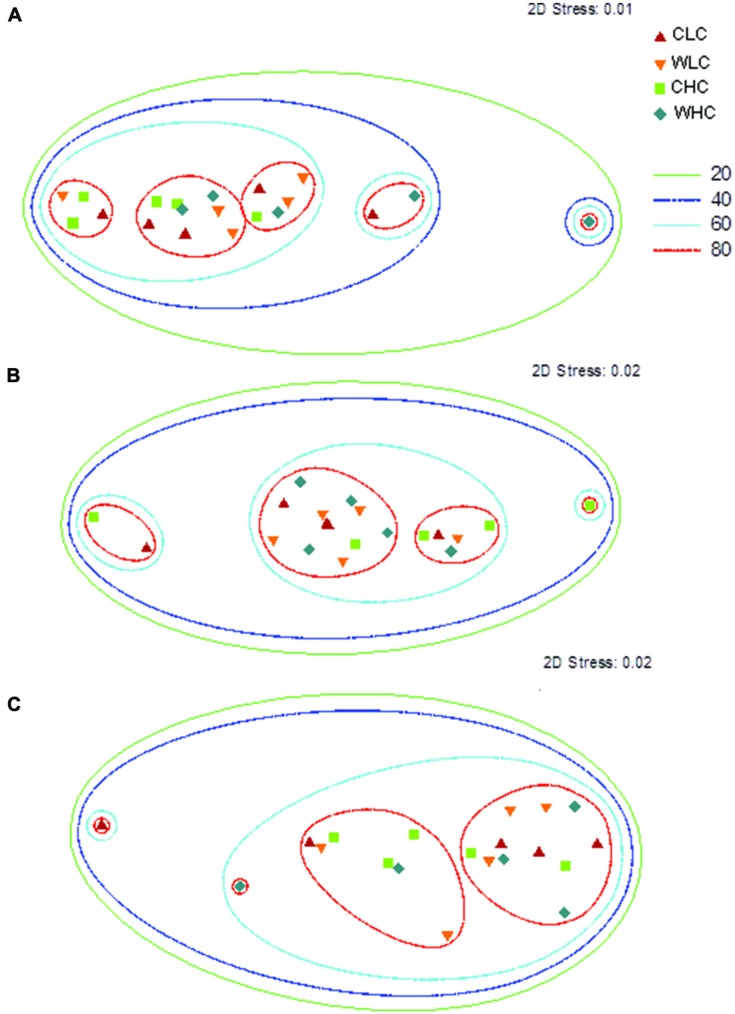
**Non-metric multi-dimensional scaling (NMDS) ordination of the PLFA data at the beginning **(A)**, and 16 **(B)** and 53 **(C)** months after the beginning of the experiment**. CLC, control low biocrust cover, WLC, warming low biocrust cover, CHC, control high biocrust cover, WHC, warming high biocrust cover. The degree of similarity among the different samples is indicated by the contour lines.

**FIGURE 3:**
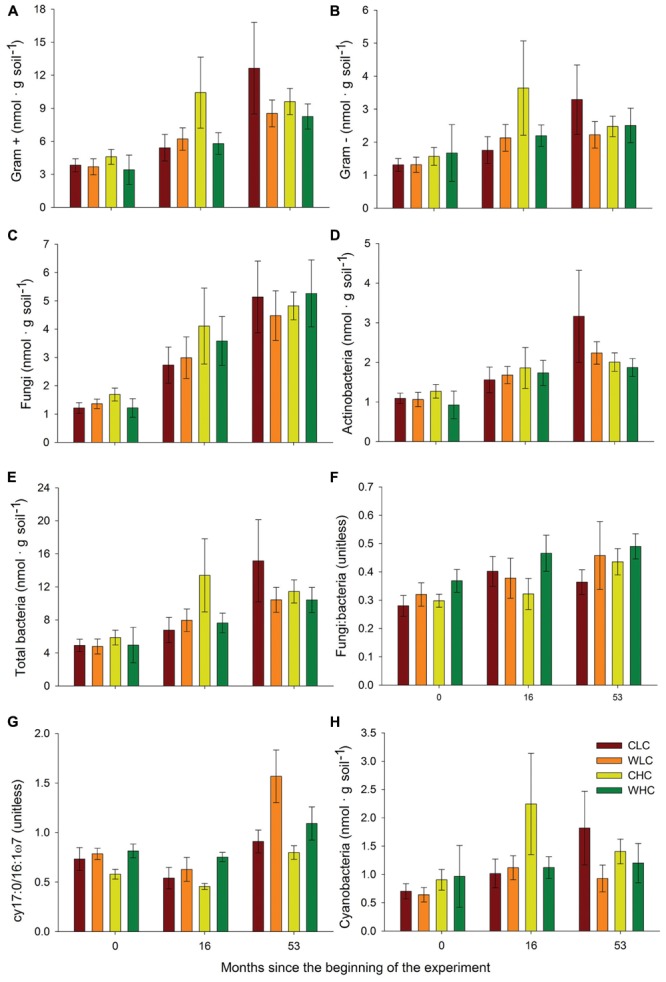
**Abundance of Gram + **(A)** and Gram — **(B)** bacteria, fungi **(C)**, actinobacteria **(D)**, total bacteria **(E)**, fungi: bacteria ratio **(F)**, cy17:0/16:1ω7 ratio **(G)** and abundance of cyanobacteria **(H)** in areas with initial low and high biocrust cover at 0, 16, and 53 months after the beginning of the experiment**. CLC, control low biocrust cover, WLC, warming low biocrust cover, CHC, control high biocrust cover, WHC, warming high biocrust cover. Data represent means and SEs (*n* = 10, 5, and 5 for 0, 16, and 53 months after the beginning of the experiment, respectively).

**FIGURE 4:**
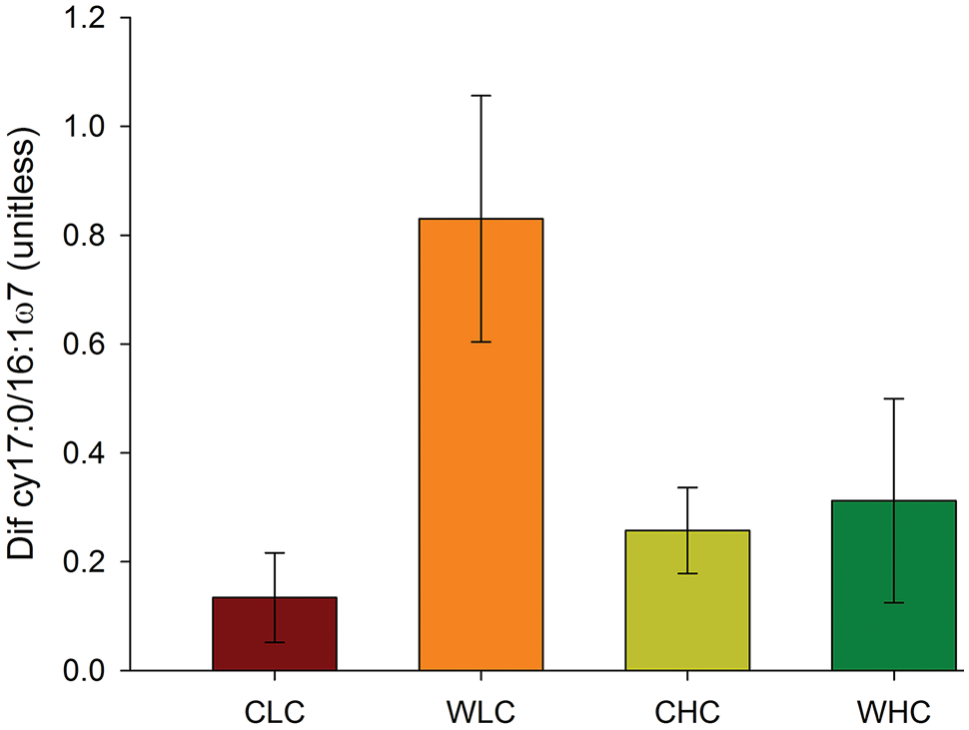
**Differences in the cy17:0/16:1ω7 ratio in plots with initial low and high biocrust cover between February 2009 and June 2013**. CLC, control low biocrust cover, WLC, warming low biocrust cover, CHC, control high biocrust cover, WHC, warming high biocrust cover. Data represent means + SE (*n* = 5).

Permutational multivariate analysis of variance analyses did not reveal significant differences among treatments in microbial PLFA composition in any of the sampling periods evaluated (*P* > 0.130 in all cases, Supplementary Table S2). This was reflected in the NMDS ordination, which showed a substantial overlap among treatments (**Figure 2**). We found a trend of increasing abundance of fatty acids associated with most microbial groups (Gram positive bacteria, Gram negative bacteria, fungi, total bacteria and actinobacteria) over the experimental period (**Figure 3**). However, no significant effects of CO and WA were found on any of the fatty acid biomarkers of these microbial groups at any of the sampling times (ANOVA, *P* > 0.134 in all cases, Supplementary Table S3), except for the cy17:0/16:1ω7 ratio, which increased with warming 16 and 53 months after the beginning of the experiment (ANOVA, *P* < 0.039 in both cases; Supplementary Table S3). To further evaluate whether biocrusts affected the responses of this ratio to warming during the course of the experiment, we obtained the Dif for the cy17:0/16:1ω7 ratio, which was analyzed with a two-way ANOVA, with WA and CO as fixed factors. These analyses revealed a marginally significant WA × CO interaction (**Figure 4**; ANOVA, *F*_1,16_ = 4.14, *P* = 0.059). Separate one-way ANOVAs showed that the increase in the cy17:0/16:1ω7 ratio with time was significant only in LC plots (LC plots, *F*_WA_ = 8.36, df = 1,8, *P* = 0.020; HC plots, *F*_WA_ = 0.07, df = 1,8, *P* = 0.794).

## Discussion

Temporal changes in the cover and richness of biocrusts were dependent on their degree of development at the beginning of the experiment. In the absence of warming, significant declines in the cover and richness of biocrusts were observed in the high cover plots over the course of the experiment, which mimics what has been reported in other biocrust-dominated ecosystems. For example, Dettweiler-Robinson et al. (2013a) found that the total cover of visible biocrust components was reduced by ∼30% over a period of 10 years in south–central Washington (USA). These results likely reflect the successional dynamics of the biocrusts studied, which are driven by both abiotic (such as fluctuations in climate; Belnap et al., 2006) and biotic (such as competition for space; Maestre et al., 2008; Bowker et al., 2010; Dettweiler-Robinson et al., 2013b) factors. The decline in the cover and richness of biocrusts observed was exacerbated by warming, which promoted a significant reduction in the cover and richness of well-developed biocrust communities, an effect that was mainly due to the response of their constituent lichens. This finding is consistent with other investigations of warming effects on biocrust-dominated grasslands and shrublands (Escolar et al., 2012; Maestre et al., 2013), and with those of Belnap et al. (2006), who reported that a 6°C increase in maximum summer temperatures over 8 years substantially reduced the cover of biocrust-forming lichens in the Colorado Plateau. Declines in biocrust cover with warming were paralleled by reductions in species richness, as also found in other studies in central Spain (Escolar et al., 2012) and the Arctic (Wahren et al., 2005; Lang et al., 2012). The main species that decreased their frequency with warming were the lichens *Diploschistes diacapsis*, *Fulgensia subbracteata*, *Toninia sedifolia* and *Placidium squamulosum* (Supplementary Table S1). Although our measurements cannot be used to identify the mechanisms underlying the observed reductions in the cover and richness of lichens, we speculate that they are promoted by the negative effects of warming on their photosynthetic activity, a response already observed with warming experiments in South Africa (Maphangwa et al., 2012) and Spain (Maestre et al., 2013; Ladrón de Guevara et al., 2014). We suggest that these effects of warming are not caused by the increase of temperature *per se* induced by this treatment, but rather by the negative effects of warming on the duration and intensity of dew events, i.e., periods with relative air humidity of 100% (Maphangwa et al., 2012; Maestre et al., 2013; Ladrón de Guevara et al., 2014). In our study area, dew events occurred in 85% of the days, and our warming treatment reduced their duration by 44% on average (Supplementary Figure S4). Moisture inputs by dew are critical for maintaining the hydration status and metabolic activity of biocrust-forming lichens such as studied (Veste et al., 2001; del Prado and Sancho, 2007; Rao et al., 2009; Maphangwa et al., 2012). Thus, it is likely that the effects of warming on these water inputs drove the reductions in the cover and richness of biocrust-forming lichens observed in our experiment. It is important to note that in the future, increased temperatures, such as those simulated in our experiment, will be combined with higher CO_2_ concentrations, which have been found to enhance the photosynthetic activity of lichens, including those studied here (Lane et al., 2013). Whether this enhancement of photosynthesis can compensate the reduced moisture inputs that are expected in a warmer climate is a topic of great interest that needs to be addressed in future studies.

The strong negative effects of warming on the cover of lichens were not found for mosses. These findings are similar to those found in a semi-arid *Stipa tenacissima* steppe in central Spain (Escolar et al., 2012), and to those reported by Reed et al. (2012) and Zelikova et al. (2012), who found very limited effects of a 2/4°C warming on these organisms. Mosses usually break their dormancy during the favorable season in terms of humidity and soil moisture (Kappen and Valladares, 2007; Bjerke et al., 2011). This could occur inside the warming plots during autumn and early winter, when temperature and moisture are adequate for the development of new stems and the reactivation of the activity of mosses. It is also likely that the responses observed with mosses are due to their high plasticity to adapt to changes in temperature and desiccation regimes (Green et al., 2011).

Phospholipid fatty acid concentrations in soils are function of the accumulated microbial response to environmental change, which may cause a change in the number of microbial cells expressing specific PLFA that are indicative of specific groups (Andresen et al., 2014). Our warming treatment had no detectable effect on the abundance or composition of major microbial groups under biocrusts determined using biomarker PLFA, regardless of the degree of development of the biocrust community. Previous climate change studies conducted with biocrust-associated microbial communities in drylands have so far reported contrasting results. For instance, Zelikova et al. (2012) found that a 2°C warming reduced both bacterial and fungal biomass after one growing season in the Colorado Plateau (USA), and Maestre et al. (2013) and Delgado-Baquerizo et al. (2014) reported increases in the fungal: bacterial ratio with warming under biocrusts in central Spain. However, Johnson et al. (2012) did not observe any effect of a 2°C soil warming on the composition of the bacterial community after 2 years. Similarly, Yeager et al. (2012) reported that a 2–3°C increase in soil temperature did not modify the diazotroph community structure over the same period. The overall lack of responses of the soil microbial communities to warming in our experiment suggests that the temperature increase induced by this treatment did not alter the normal temperature range experienced by these organisms (*sensu* Pereira-Silva et al., 2011). Furthermore, if we consider that the temperature is expected to increase gradually over time in response to increases in atmospheric concentrations of CO_2_ and other greenhouse gasses, it is likely that the studied microbes will have enough time for adapt to the future temperatures (Bradford, 2013). However, we may not discard the possibility that the resolution of analysis using PLFA is insufficient to detect any possible taxonomic changes in the different microbial groups studied with warming in the study area. Due to the lack of phylogenetic resolution of PLFA, we cannot discard the possibility that microbial taxa will have been affected by warming, as has been reported in previous experiments (Liang et al., 2015).

The physiological stress ratio of PLFA, cy17:0/16:1ω7, is an example of how microbial responses to changes in environmental factors such as water, nutrient and temperature may also modify the biochemistry of the microbial membranes of individual bacteria (Petersen and Klug, 1994; Bossio and Scow, 1998; Kaur et al., 2005; Andresen et al., 2014). As 16:1ω7 and cy17:0 are Gram negative biomarkers, this ratio indicates the degree of physiological stress experience by the Gram negative bacterial community; increases in this index typically indicate a starvation response or a shift to stationary phase growth in Gram negative bacteria (Ramos et al., 2001). We observed that warming increased the cy17:0/16:1ω7 ratio 16 and 53 months after the beginning of the experiment, suggesting an alteration of the physiological status of the soil microbial communities. Ben-David et al. (2011) also reported that the cy17:0/16:1ω7 ratio was higher in the open areas dominated by biocrust communities than under the canopy of shrubs, as well as in an arid site relative to a semi-arid site in Israel. These differences were likely due to the relative increases in evapotranspiration and radiation experienced by soil microorganisms under biocrust *versus* vegetation (as well as in the arid *versus* semi-arid sites; Ben-David et al., 2011). In our experiment, warming reduced both soil moisture and the duration of dew events particularly under periods of high ambient moisture conditions (Supplementary Figures S3 and S4), which correspond to those where semi-arid Mediterranean biocrust-forming lichens are most active (Pintado et al., 2010; Ladrón de Guevara et al., 2014). The reduction in biocrust activity and declines in cover with warming probably reduced the inputs of labile carbon and nutrient inputs to the soil over the course of our experiment, as well as the formation of a matrix of extracellular exopolysaccharides produced by biocrust-forming cyanobacteria (Colica et al., 2014). This, together with the microclimatic changes associated to the losses in biocrust cover, may have the increased degree of stress experienced by the Gram negative bacteria (e.g., Bossio and Scow, 1998; Lundquist et al., 1999; Ramos et al., 2001; Fierer et al., 2003; Brant et al., 2006). However, we did not observe a coincident change in the composition and abundance of the major microbial groups, i.e., a shift to a stress-tolerator community dominated by *K*-strategists (actinobacteria and fungi; Dungait et al., 2011; Dungait et al., 2013). As noted above, it is plausible that the resolution of PLFA did not capture changes in the phylogenetic and functional diversity of soil microbial communities with warming. Alternatively, drying and rewetting is an example of a major stress which challenges soil microorganisms in dryland systems (Placella et al., 2012), so the microbial groups studied may have already been stress-tolerant and therefore slow to respond to relatively minor temperature changes. In the longer term a change may have been observed. A chronosequence (5, 8, and 20 years) of soil warming (5°C) experiments in the Harvard Forest (USA) detected only shifts in the composition of soil microbial communities 20 years after commencing treatment (DeAngelis et al., 2015). Regardless of the mechanism, our findings indicate that warming increased the degree of stress experienced by the Gram negative bacterial community associated with biocrusts during the first years.

While the effects of warming on the cy17:0/16:1ω7 ratio were evident when analyzing the samples collected at particular time points, an evaluation of the differences in this ratio over the course of the experiment revealed a significant biocrust × warming interaction, as increases in this ratio through time were significant only in LC, but not in HC plots (**Figure 4**). These results suggest that biocrusts provide resistance, defined as “the amount of change caused by a disturbance” (Orwin and Wardle, 2004), against the effects of warming on the degree of physiological stress experienced by the soil microbial communities. Similarly to what we found with the cy17:0/16:1ω7 ratio, Delgado-Baquerizo et al. (2014) reported that lichen-dominated biocrusts increased the resistance to warming of multiple variables linked to soil nitrogen availability in an experiment conducted in central Spain. Although we cannot provide a mechanistic explanation for our findings, they may be driven by the effects of biocrusts on the availability of resources for microorganisms, and on the way biocrusts affect the response to these resources to warming. As found in other studies conducted with biocrusts elsewhere (Maestre et al., 2013; Miralles et al., 2013), HC plots had higher organic carbon contents than LC plots (Cristina Escolar, unpublished data), a response likely driven by the carbon inputs derived from the photosynthetic activity of biocrust constituents (Li et al., 2012; Huang et al., 2014). A recent study conducted with biocrust communities similar to that we studied revealed those HC plots showed higher water gains and slower water loses than LC plots after rainfall events, which led to constituently higher soil moisture values in the former over a six–year (Berdugo et al., 2014). In our experiment, reductions in soil moisture with warming were more evident in the LC than in the HC plots over the course of our experiment (Supplementary Figure S5). Thus, increased carbon and water availability under biocrusts could have reduced the degree of stress experienced by the microbial communities with warming (Bossio and Scow, 1998; Lundquist et al., 1999; Brant et al., 2006). Our findings complement those from recent climate change studies conducted with biocrusts (Reed et al., 2012; Maestre et al., 2013; Delgado-Baquerizo et al., 2014), and highlight the importance of these organisms to understand microbial responses to climate change drivers in drylands.

In summary, we found that 53 months of experimental warming significantly reduced both the richness and cover of lichen-dominated biocrusts in the semi-arid plantation studied, but had only limited impacts on associated soil microbial communities, as measured by PLFA analysis. The observed increase in the cy17:0/16:1ω7 ratio, an indicator of the physiological stress of Gram negative bacteria, through time induced by warming was only detected in the absence of biocrusts. Our findings suggest that amelioration of soil and microclimatic conditions provided by biocrusts can slow down the negative effects of warming on the physiological status of Gram negative soil bacteria. However, the negative impacts of warming on the cover and richness of biocrusts will limit their positive impacts on the physiological status of these soil bacterial communities under a warmer climate.

## Author Contributions

FM designed the experiment; CE, VO, and BG conducted field and laboratory analyses; CE, JD, and RB conducted PLFA analyses; FM and CE analyzed data; FM wrote the paper, with substantial inputs from RB, JD and CE.

## Conflict of Interest Statement

The authors declare that the research was conducted in the absence of any commercial or financial relationships that could be construed as a potential conflict of interest.

## Footnotes

1 http://www.gimp.org/

2 http://rsb.info.nih.gov/ij/.

## References

[B1] Anderson M. J. (2001). A new method for non-parametric multivariate analysis of variance. *Austral. Ecol.* 26 32–46. 10.1111/j.1442-9993.2001.01070.pp.x

[B2] Anderson M. J. Ter Braak C. J. F. (2003). Permutation tests for multi-factorial analysis of variance. *J. Stat. Comput. Simul.* 73 85–113. 10.1080/00949650215733

[B3] Anderson M. J. Walsh D. C. I. (2013). PERMANOVA, ANOSIM, and the Mantel test in the face of heterogeneous dispersions: what null hypothesis are you testing? *Ecol. Monogr.* 83 557–574. 10.1890/12-2010.1

[B4] Andresen L. C. Dungait J. A. J. Bol R. Selsted M. B. Ambus P. Michelsen A. (2014). Bacteria and fungi respond differently to multifactorial climate change in a temperate heathland, traced with 13C-Glycine and FACE CO_2_. *PLoS ONE* 9:e85070 10.1371/journal.pone.0085070 PMC3893180 24454793

[B5] Arft A. M. Walker M. D. Gurevitch J. Alatalo J. M. Bret-Harte M. S. Dale M. (1999). Responses of tundra plants to experimental warming: meta-analysis of the international tundra experiment. *Ecol. Monogr.* 69 491–511. 10.2307/2657227

[B6] Bárcenas-Moreno G. García-Orenes F. Mataix-Beneyto J. Bååth E. (2014). Plant species influence on soil microbial short-term response after fire simulation. *Plant. Soil* 374 701–713. 10.1007/s11104-013-1889-4

[B7] Bardgett R. D. Hobbs P. J. Frostegård A. (1996). Changes in soil fungal: bacterial biomass ratios following reductions in the intensity of management of an upland grassland. *Biol. Fertil. Soils* 22 261–264. 10.1007/s003740050108

[B8] Barger N. N. Castle S. C. Dean G. N. (2013). Denitrification from nitrogen-fixing biologically crusted soils in a cool desert environment, southeast Utah, USA. *Ecol. Process* 2 16 10.1186/2192-1709-2-16

[B9] Barrios E. (2007). Soil biota, ecosystem services and land productivity. *Ecol. Econ.* 64 269–285. 10.1016/j.ecolecon.2007.03.004

[B10] Bates S. T. Nash Thomas H., Sweata K. G. Garcia-Pichel F. (2010). Fungal communities of lichen-dominated biological soil crusts: diversity, relative microbial biomass, and their relationship to disturbance and crust cover. *J. Arid. Environ.* 74 1192–1199. 10.1016/j.jaridenv.2010.05.033

[B11] Belnap J. (2003). The world at your feet: desert biological soil crusts. *Front. Ecol. Environ* 1:181–189. 10.1890/1540-9295(2003)001[0181:TWAYFD]2.0.CO;2

[B12] Belnap J. Lange O. L. (2001). *Biological Soil Crusts: Structure, Function, and Management.* Berlin: Springer-Verlag.

[B13] Belnap J. Phillips S. Troxler T. (2006). Soil lichen and moss cover and species richness can be highly dynamic: the effects of invasion by the annual exotic grass *Bromus tectorum*, precipitation, and temperature on biological soil crusts in SE Utah. *Appl. Soil Ecol.* 32 63–76. 10.1016/j.apsoil.2004.12.010

[B14] Ben-David E. A. Zaady E. Sher Y. Nejidat A. (2011). Assessment of the spatial distribution of soil microbial communities in patchy arid and semi-arid landscapes of the Negev Desert using combined PLFA and DGGE analyses. *FEMS Microbiol. Ecol.* 76 492–503. 10.1111/j.1574-6941.2011.01075.x 21401693

[B15] Berdugo M. Soliveres S. Maestre F. T. (2014). Vascular plants and biocrusts modulate how abiotic factors affect wetting and drying events in drylands. *Ecosystems* 17 1242–1256. 10.1007/s10021-014-9790-4

[B16] Bjerke J. W. Bokhorst S. Zielke M. Callaghan T. V. Bowles F. W. Phoenix G. K. (2011). Contrasting sensitivity to extreme winter warming events of dominant sub-Arctic heathland bryophyte and lichen species. *J. Ecol.* 99 1481–1488. 10.1111/j.1365-2745.2011.01859.x

[B17] Bodelier P. L. E. Gillisen M.-J. B. Hordijk K. Damsté J. S. S. Rijpstra W. I. C. Geenevasen J. A. J. (2009). A reanalysis of phospholipid fatty acids as ecological biomarkers for methanotrophic bacteria. *ISME J.* 3 606–617. 10.1038/ismej.2009.6 19194481

[B18] Bossio D. A. Scow K. A. (1998). Impacts of carbon and flooding on soil microbial communities: phospholipid fatty acid profiles and substrate utilization patterns. *Microb. Ecol.* 35 265–278. 10.1007/s002489900082 9569284

[B19] Bowker M. A. Mau R. L. Maestre F. T. Escolar C. Castillo-Monroy A. P. (2011). Functional profiles reveal unique ecological roles of various biological soil crust organisms. *Funct. Ecol.* 25 787–795. 10.1111/j.1365-2435.2011.01835.x

[B20] Bowker M. A. Soliveres S. Maestre F. T. (2010). Competition increases with abiotic stress and regulates the diversity of biological soil crusts. *J. Ecol.* 98 551–560. 10.1111/j.1365-2745.2010.01647.x

[B21] Bradford M. A. (2013). Thermal adaptation of decomposer communities in warming soils. *Front. Microbiol.* 4:1–16. 10.3389/fmicb.2013.00333 24339821 PMC3825258

[B22] Brant J. B. Myrold D. D. Sulzman E. W. (2006). Root controls on soil microbial community structure in forest soils. *Oecologia* 148 650–659. 10.1007/s00442-006-0402-7 16547734

[B23] Brussaard L. (2012). “Ecosystem services provided by the soil biota,” in *Soil Ecology Ecosystems Services*, eds Wall D. H. Bardgett R. D. Behan-Pelletier V. Herrick J. E. Jones T. H. Ritz K. (Oxford: Oxford University Press) 201–232.

[B24] Buitenwerf R. Rose L. Higgins S. I. (2015). Three decades of multi-dimensional change in global leaf phenology. *Nat. Clim. Change* 5 364–368. 10.1038/nclimate2533

[B25] Castillo-Monroy A. P. Bowker M. A. Maestre F. T. Rodríguez-Echeverría S. Martinez I. Barraza-Zepeda C. E. (2011a). Relationships between biological soil crusts, bacterial diversity and abundance, and ecosystem functioning: insights from a semi-arid Mediterranean environment. *J. Veg. Sci.* 22 165–174. 10.1111/j.1654-1103.2010.01236.x

[B26] Castillo-Monroy A. P. Maestre F. T. Rey A. Soliveres S. García-Palacios P. (2011b). Biological soil crust microsites are the main contributor to soil respiration in a semiarid ecosystem. *Ecosystems* 14 835–847. 10.1007/s10021-011-9449-3

[B27] Castillo-Monroy A. P. Maestre F. T. Delgado-Baquerizo M. Gallardo A. (2010). Biological soil crusts modulate nitrogen availability in semi-arid ecosystems: insights from a Mediterranean grassland *Plant. Soil* 333 21–34. 10.1007/s11104-009-0276-7

[B28] Colica G. Li H. Rossi F. Li D. Liu Y. De Philippis R. (2014). Microbial secreted exopolysaccharides affect the hydrological behavior of induced biological soil crusts in desert sandy soils. *Soil Biol. Biochem.* 68 62–70. 10.1016/j.soilbio.2013.09.017

[B29] Concostrina-Zubiri L. Huber-Sannwald E. Martínez I. Flores Flores J. L. Escudero A. (2013). Biological soil crusts greatly contribute to small-scale soil heterogeneity along a grazing gradient. *Soil Biol. Biochem.* 64 28–36. 10.1016/j.soilbio.2013.03.029

[B30] DeAngelis K. M. Pold G. Topçuoðlu B. D. van Diepen L. T. A. Varney R. M. Blanchard J. L. (2015). Long-term forest soil warming alters microbial communities in temperate forest soils. *Front. Microbiol.* 6:104 10.3389/fmicb.2015.00104 PMC4327730 25762989

[B31] De Castro M. Martín-Vide J. Alonso S. (2005). *El Clima de España: Pasado, Presente y Escenarios de Clima Para el Siglo XXI. Impactos del Cambio Climático en España.* Madrid: Ministerio Medio Ambiente.

[B32] del Prado R. Sancho L. G. (2007). Dew as a key factor for the distribution pattern of the lichen species *Teloschistes lacunosus* in the Tabernas Desert (Spain). *Flora* 202 417–428. 10.1016/j.flora.2006.07.007

[B33] Delgado-Baquerizo M. Escolar C. Gallardo A. Ochoa V. Gozalo B. Prado-Comesaña A. (2014). Direct and indirect impacts of climate change on microbial and biocrust communities alter the resistance of the N cycle in a semiarid grassland. *J. Ecol.* 102 1592–1605. 10.1111/1365-2745.12303

[B34] Dettweiler-Robinson E. Ponzetti J. M. Bakker J. D. (2013a). Long-term changes in biological soil crust cover and composition. *Ecol. Process.* 2:5 10.1186/2192-1709-2-5

[B35] Dettweiler-Robinson E. Bakker J. D. Grace J. B. (2013b). Controls of biological soil crust cover and composition shift with succession in sagebrush shrub-steppe. *J. Arid Environ.* 94 96–104. 10.1016/j.jaridenv.2013.01.013

[B36] Dungait J. A. J. Kemmitt S. J. Michallon L. Guo S. Wen Q. Brookes P. C. (2011). Variable responses of the soil microbial biomass to trace concentrations of 13C-labelled glucose, using 13C-PLFA analysis. *Eur. J. Soil Sci.* 62 117–126. 10.1111/j.1365-2389.2010.01321.x

[B37] Dungait J. A. J. Kemmitt S. J. Michallon L. Guo S. Wen Q. Brookes P. C. (2013). The variable response of soil microorganisms to trace concentrations of low molecular weight organic substrates of increasing complexity. *Soil Biol. Biochem.* 64 57–64. 10.1016/j.soilbio.2013.03.036

[B38] Elbert W. Weber B. Burrows S. Steinkamp J. Büdel B. Andreae M. O. (2012). Contribution of cryptogamic covers to the global cycles of carbon and nitrogen. *Nat. Geosci.* 5 459–462. 10.1038/ngeo1486

[B39] Eldridge D. J. Greene R. S. B. (1994). Microbiotic soil crusts: a review of their roles in soil and ecological processes in the rangelands of Australia. *Aust. J. Soil Res.* 32 389–415. 10.1071/SR9940389

[B40] Escolar C. Martínez I. Bowker M. A. Maestre F. T. (2012). Warming reduces the growth and diversity of biological soil crusts in a semi-arid environment: implications for ecosystem structure and functioning. *Philos. Trans. R. Soc. Lond. B* 367 3087–3099. 10.1098/rstb.2011.03441 23045707 PMC3479686

[B41] Federle T. W. (1986). “Microbial distribution in soil new techniques,” in *Perspectives in Microbial Ecology*, eds Megusar F. Ganthar M. (Ljubljana: Slovene Society for Microbiology) 493–498.

[B42] Fierer N. Jackson R. B. (2006). The diversity and biogeography of soil bacterial communities. *Proc. Natl. Acad. Sci. U.S.A.* 103 626–631. 10.1073/pnas.0507535103 16407148 PMC1334650

[B43] Fierer N. Schimel J. P. Holden P. A. (2003). Variations in microbial community composition through two soil depth profiles. *Soil Biol. Biochem.* 35 167–176. 10.1016/S0038-0717(02)00251-1

[B44] Fitter A. H. Gilligan C. A. Hollingworth K. Kleczkowski A. Twyman R. M. Pitchford J. W. (2005). Biodiversity and ecosystem function in soil. *Funct. Ecol.* 19 369–377. 10.1111/j.0269-8463.2005.00969.x

[B45] Frostegård A. Bååth E. (1996). The use of phospholipid analysis to estimate bacterial and fungal biomass in soils. *Biol. Fert. Soils.* 22 59–65. 10.1007/BF00384433

[B46] Frostegård A. Tunlid A. Bååth E. (1991). Microbial biomass measured as total lipid phosphate in soils of different organic content. *J. Microbiol. Meth.* 14 151–163. 10.1016/0167-7012(91)90018-L

[B47] Frostegård A. Tunlid A. Bååth E. (1993). Phospholipid fatty acid composition, biomass, and activity of microbial communities from two soil types experimentally exposed to different heavy metals. *Appl. Environ. Microbiol.* 59 3605–3617. 16349080 10.1128/aem.59.11.3605-3617.1993 PMC182506

[B48] Frostegård, Å Tunlid A. Bååth E. (2011). Use and misuse of PLFA measurements in soils. *Soil Biol. Biochem.* 43 1621–1625. 10.1016/j.soilbio.2010.11.021

[B49] Garcia-Pichel F. Loza V. Marusenko Y. Mateo P. Potrafka R. M. (2013). Temperature drives the continental-scale distribution of key microbes in topsoil communities. *Science* 340 1574–1577. 10.1126/science.1236404 23812714

[B50] Green T. G. A. Sancho L. G. Pintado A. (2011). “Ecophysiology of desiccation/rehydration cycles in mosses and lichens,” in *Plant Desiccation Tolerance, Part 2, Ecological Studies 215*, eds Luttge U. Beck E. Bartels D. (Berlin: Springer-Verlag) 89–120.

[B51] Grote E. E. Belnap J. Housman D. C. Sparks J. P. (2010). Carbon exchange in biological soil crust communities under differential temperatures and soil water contents: implications for global change. *Glob. Change Biol.* 16 2763–2774. 10.1111/j.1365-2486.2010.02201.x

[B52] Guckert J. B. Hood M. A. White D. C. (1986). Phospholipid ester-linked fatty acid profile changes during nutrient deprivation of *Vibrio cholerae*: increases in the trans/cis ratio and proportions of cyclopropyl fatty acids. *Appl. Environ. Microbiol.* 52 794–801. 3777927 10.1128/aem.52.4.794-801.1986 PMC239116

[B53] Hartley I. P. Garnett M. H. Sommerkorn M. Hopkins D. W. Fletcher B. J. Sloan V. L. (2012). A potential loss of carbon associated with greater plant growth in the European Arctic. *Nat. Clim. Change* 2 875–879. 10.1038/nclimate1575

[B54] Housman D. C. Yeager C. M. Darby B. J. Sanford R. L. Kuske C. R. Neher D. A. (2007). Heterogeneity of soil nutrients and subsurface biota in a dryland ecosystem. *Soil Biol. Biochem.* 39 2138–2149. 10.1016/j.soilbio.2007.03.015

[B55] Huang L. Zhang Z. Li X. (2014). Carbon fixation and its influence factors of biological soil crusts in a revegetated area of the Tengger Desert, northern China. *J. Arid Land* 6 725–734. 10.1007/s40333-014-0027-3

[B56] IUSS Working Group WRB. (2006). *World Reference Base for Soil Resources 2006.* World Soil Resources Reports No. 103. FAO, Rome.

[B57] Johnson S. L. Kuske C. R. Carney T. D. Housman D. C. Gallegos-Graves L. V. Belnap J. (2012). Increased temperature and altered summer precipitation have differential effects on biological soil crusts in a dryland ecosystem. *Glob. Change Biol.* 18 2583–2593. 10.1111/j.1365-2486.2012.02709.x

[B58] Kappen L. Valladares F. (2007). “Opportunistic growth and desiccation tolerance: the ecological success of poikilohydrous autotrophs,” in *Functional Plant Ecology*, eds Pugnaire F. Valladares F. (New York, NY: Taylor and Francis Group) 7–65.

[B59] Kaur A. Chaudhary A. Kaur A. Choudhary R. Kaushik R. (2005). Phospholipid fatty acid. A bioindicator of environment monitoring assessment in soil ecosystem. *Curr. Sci.* 89 1103–1112.

[B60] Ladrón de Guevara M. Lázaro R. Quero J. L. Ochoa V. Gozalo B. Berdugo M. (2014). Simulated climate change reduced the capacity of lichen-dominated biocrusts to act as carbon sinks in two semi-arid Mediterranean ecosystems. *Biodivers. Conserv.* 23 1787–1807. 10.1007/s10531-014-0681-y

[B61] Lane R. W. Menon M. McQuaid J. B. Adams D. G. Thomas A. D. Hoon S. R. (2013). Laboratory analysis of the effects of elevated atmospheric carbon dioxide on respiration in biological soil crusts. *J. Arid Environ.* 98 52–59. 10.1016/j.jaridenv.2013.07.014

[B62] Lang S. I. Cornelissen J. H. C. Shaver G. R. Ahrens M. Callaghan T. V. Molau U. (2012). Arctic warming on two continents has consistent negative effects on lichen diversity and mixed effects on bryophyte diversity. *Glob. Change. Biol.* 18 1096–1107. 10.1111/j.1365-2486.2011.02570.x

[B63] Li X. R. Zhang P. Su Y. G. Jia R. L. (2012). Carbon fixation by biological soil crusts following revegetation of sand dunes in arid desert regions of China: a four-year field study. *Catena* 97 119–126. 10.1016/j.catena.2012.05.009

[B64] Liang Y. Jiang Y. Wang F. Wen C. Deng Y. Xue K. (2015). Long-term soil transplant simulating climate change with latitude significantly alters microbial temporal turnover. *ISME J.* 10.1038/ismej.2015.78 PMC4817637 25989371

[B65] Lundquist E. J. Scow K. M. Jackson L. E. Uesugi S. L. Johnson C. R. (1999). Rapid response of soil microbial communities from convertional low input, and organic farming systems to a wet/dry cycle. *Soil Biol. Biochem.* 31 1661–1675. 10.1016/S0038-0717(99)00080-2

[B66] Maestre F. T. (2000). *El Medio Ambiente en Sax. Estado Actual y Propuestas de Gestión.* Alicante: Universidad de Alicante y Ayuntamiento de Sax.

[B67] Maestre F. T. Bowker M. A. Cantón Y. Castillo-Monroy A. P. Cortina J. Escolar C. (2011). Ecology and functional roles of biological soil crusts in semi-arid ecosystems of Spain. *J. Arid Environ.* 75 1282–1291. 10.1016/j.jaridenv.2010.12.008 25908884 PMC4404999

[B68] Maestre F. T. Escolar C. Bardgett R. Dungait J. A. D. Gozalo B. Ochoa V. (2015). Data from “Warming reduces the cover and diversity of biocrust-forming mosses and lichens, and increases the physiological stress of soil microbial communities in a semi-arid *Pinus halepensis* plantation.” *figshare*10.6084/m9.figshare.1486354 PMC4548238 26379642

[B69] Maestre F. T. Escolar C. de Guevara M. L. Quero J. L. Lázaro R. Delgado-Baquerizo M. (2013). Changes in biocrust cover drive carbon cycle responses to climate change in drylands. *Glob. Change Biol.* 19 3835–3847. 10.1111/gcb.12659 PMC3942145 23818331

[B70] Maestre F. T. Escolar C. Martínez I. Escudero A. (2008). Are soil lichen communities structured by biotic interactions? A null model analysis. *J. Veg. Sci.* 19 261–266. 10.3170/2007-8-18366

[B71] Maestre F. T. Escudero A. Martínez I. Guerrero C. Rubio A. (2005). Does spatial pattern matter to ecosystem functioning? Insights from biological soil crusts. *Funct. Ecol.* 19 566–573. 10.1111/j.1365-2435.2005.01000.x

[B72] Makhalanyane T. P. Valverde A. Gunnigle E. Frossard A. Ramond J.-B. Cowan D. (2015). Microbial ecology of hot desert edaphic systems. *FEMS Microbiol. Rev.* 39 203–221. 10.1093/femsre/fuu011 25725013

[B73] Maphangwa K. W. Musil C. F. Raitt L. Zedda L. (2012). Experimental climate warming decreases photosynthetic efficiency of lichens in an arid South African ecosystem. *Oecologia* 169 257–268. 10.1007/s00442-011-2184-9 22057927

[B74] Miralles I. Trasar-Cepeda C. Leirós M. C. Gil-Sotres F. (2013). Labile carbon in biological soil crusts in the Tabernas desert, SE Spain. *Soil Biol. Biochem.* 58 1–8. 10.1016/j.soilbio.2012.11.010

[B75] Mortillaro J.-M. Rigal F. Rybarczyk H. Bernardes M. Abril G. Meziane T. (2012). Particulate organic matter distribution along the lower Amazon River: addressing aquatic ecology concepts using fatty acids. *PLoS ONE* 7:e46141 10.1371/journal.pone.0046141 PMC3460950 23029412

[B76] Orwin K. H. Wardle D. A. (2004). New indices for quantifying the resistance and resilience of soil biota to exogenous disturbances. *Soil Biol. Biochem.* 36 1907–1912. 10.1016/j.soilbio.2004.04.036

[B77] Pasternak Z. Al-Ashhab A. Gatica J. Gafny R. Avraham S. Minz D. (2013). Spatial and temporal biogeography of soil microbial communities in arid and semiarid regions. *PLoS ONE* 8:e69705 10.1371/journal.pone.0069705 PMC3724898 23922779

[B78] Peñuelas J. Sardans J. Estiarte M. Ogaya R. Carnicer J. Coll M. (2013). Evidence of current impact of climate change on life: a walk from genes to the biosphere. *Glob. Change Biol.* 19 2303–2338. 10.1111/gcb.12143 23505157

[B79] Pereira-Silva M. C. Semenov A. V. van Elsas J. D. Salles J. F. (2011). Seasonal variations in the diversity and abundance of diazotrophic communities across soils. *FEMS Microbiol. Ecol.* 77 57–68. 10.1111/j.1574-6941.2011.01081.x 21385188

[B80] Petersen S. O. Klug M. J. (1994). Effects of Sieving, storage, and incubation temperature on the phospholipid fatty acid profile of a soil microbial community. *Appl. Environ. Microbiol.* 60 2421–2430. 16349325 10.1128/aem.60.7.2421-2430.1994 PMC201666

[B81] Pintado A. Sancho L. G. Blanquer J. M. Green T. G. A. Lázaro R. (2010). Microclimatic factors and photosynthetic activity of crustose lichens from the semiarid southeast of Spain: long-term measurements for *Diploschistes diacapsis*. *Bibl. Lichenol.* 105 211–224.

[B82] Placella S. A. Brodie E. L. Firestone M. K. (2012). Rainfall-induced carbon dioxide pulses result from sequential resuscitation of phylogenetically clustered microbial groups. *Proc. Nat. Acad. Sci. U.S.A.* 109 10931–10936. 10.1073/pnas.1204306109 PMC3390866 22715291

[B83] Ramirez K. S. Leff J. W. Barberán A. Bates S. T. Betley J. Thomas W. (2014). Biogeographic patterns in below-ground diversity in New York City’ s Central Park are similar to those observed globally. *Proc. R. Soc. B Biol. Sci.* 281 20141988 10.1098/rspb.2014.1988 PMC4213626 25274366

[B84] Ramos J. L. Gallegos M. T. Marques S. Ramos-Gonzales M. I. Espinosa-Urgel M. Segura A. (2001). Responses of Gram-negative bacteria to certain environmental stressors. *Curr. Opin. Microbiol.* 4 166–171. 10.1016/S1369-5274(00)00183-1 11282472

[B85] Ramsey P. W. Rillig M. C. Feris K. P. Holben W. E. Gannon J. E. (2006). Choice of methods for soil microbial community analysis: PLFA maximizes power compared to CLPP and PCR-based approaches. *Pedobiologia* (*Jena*) 50 275–280. 10.1016/j.pedobi.2006.03.003

[B86] Rao B. Liu Y. Wang W. Hu C. Dunhai L. Lan S. (2009). Influence of dew on biomass and photosystem II activity of cyanobacterial crusts in the Hopq Desert, northwest China. *Soil Biol. Biochem.* 41 2387–2393. 10.1016/j.soilbio.2009.06.005

[B87] Ratledge C. Wilkinson S. G. (1988). *Microbial Lipids.* Ringelberg: Academic Press.

[B88] Reed S. C. Coe K. K. Sparks J. P. Housman D. C. Zelikova T. J. Belnap J. (2012). Changes to dryland rainfall result in rapid moss mortality and altered soil fertility. *Nat. Clim. Change* 2 752–755. 10.1038/nclimate1596

[B89] Rosenzweig C. Casassa G. Karoly D. J. Imeson A. Liu C. Menzel A. (2007). “Assessment of observed changes and responses in natural and managed systems,” in *Climate Change 2007: Impacts, Adaptation and Vulnerability. Contribution of Working Group II to the Fourth Assessment Report of the Intergovernmental Panel on Climate Change*, eds Parry M. L. Canziani O. F. Palutikof J. P. van der Linden P. J. Hanson C. E. (Cambridge: Cambridge University Press) 79–131.

[B90] Serna-Chavez H. M. Fierer N. Van Bodegom P. M. (2013). Global drivers and patterns of microbial abundance in soil. *Global Ecol. Biogeogr.* 22 1162–1172. 10.1111/geb.12070

[B91] Steinberger Y. Zelles L. Yun Q. Von Lützow M. Charles J. (1999). Phospholipid fatty acid profiles as indicators for the microbial community structure in soils along a climatic transect in the Judean Desert. *Biol. Fertil. Soils* 28 292–300. 10.1007/s003740050496

[B92] Steven B. Gallegos-Graves L. V. Belnap J. Kuske C. R. (2013). Dryland soil microbial communities display spatial biogeographic patterns associated with soil depth and soil parent material. *FEMS Microbiol. Ecol.* 86 101–113. 10.1111/1574-6941.12143 23621290

[B93] Veste M. Littmann T. Friedrich H. Breckle S. (2001). Microclimatic boundary conditions for activity of soil lichen crusts in sand dunes of the north-western Negev desert, Israel. *Flora* 196 465–474.

[B94] Visser M. E. Both C. (2005). Shifts in phenology due to global climate change: the need for a yardstick. *Proc. R. Soc. B* 272 2561–2569. 10.1098/rspb.2005.3356 PMC1559974 16321776

[B95] Wahren C.-H. A. Walker M. D. Bret-Harte M. S. (2005). Vegetation responses in Alaskan arctic tundra after 8 years of a summer warming and winter snow manipulation experiment. *Glob. Change Biol.* 11 537–552. 10.1111/j.1365-2486.2005.00927.x

[B96] White D. C. Pinkart H. C. Ringelberg A. B. (1997). “Biomass measurements: biochemical approaches,” in *Manual of Environmental Microbiology*, eds Hurst C. J. Knudson G. R. McInerney M. J. Stetzenbach L. D. Walter M. V. (Washington DC: ASM Press) 91–101.

[B97] Wilske B. Burgheimer J. Karnieli A. Zaady E. Andreae M. O. Yakir D. (2008). The CO2 exchange of biological soil crusts in a semiarid grass-shrubland at the northern transition zone of the Negev desert, Israel. *Biogeosciences* 5 1411–1423. 10.5194/bg-5-1411-2008

[B98] Yeager C. M. Kornosky J. L. Housman D. C. Grote E. E. Belnap J. Kuske C. R. (2004). Diazotrophic community structure and function in two successional stages of biological soil crusts from the Colorado Plateau and Chihuahuan Desert. *Appl. Environ. Microbiol.* 70 973–983. 10.1128/AEM.70.2.973-983.2004 14766579 PMC348917

[B99] Yeager C. M. Kuske C. R. Carney T. D. Johnson S. L. Ticknor L. O. Belnap J. (2012). Response of biological soil crust diazotrophs to season, altered summer precipitation, and year-round increased temperature in an arid grassland of the Colorado plateau, USA. *Front. Microbiol.* 3:358 10.3389/fmicb.2012.00358 PMC3468842 23087679

[B100] Zaady E. Ben-David E. A. Sher Y. Tzirkin R. Nejidat A. (2010). Inferring biological soil crust successional stage using combined PLFA, DGGE, physical and biophysiological analyses. *Soil Biol. Biochem.* 42 842–849. 10.1016/j.soilbio.2010.02.002

[B101] Zelikova T. J. Housman D. C. Grote E. D. Neher D. Belnap J. (2012). Biological soil crusts show limited response to warming but larger response to increased precipitation frequency: implications for soil processes on the Colorado Plateau. *Plant Soil* 355 265–282. 10.1007/s11104-011-1097-z

[B102] Zelles L. (1999). Fatty acid patterns of phospholipids and lipopolysaccharides in the characterisation of microbial communities in soil: a review. *Biol. Fertil. Soils* 29 111–129. 10.1007/s003740050533

[B103] Zhou J. Xue K. Xie J. Deng Y. Wu L. Cheng X. (2012). Microbial mediation of carbon-cycle feedbacks to climate warming. *Nat. Clim. Change* 2 106–110. 10.1038/nclimate1331

[B104] Zogg G. P. Zak D. R. Ringelberg D. B. Macdonald N. W. Pregitzer K. S. White D. C. (1997). Compositional and functional shifts in microbial communities due to soil Warming. *Soil Sci. Soc. Am. J.* 61 475–481. 10.2136/sssaj1997

