# Supplementary material for: Warming reduces the cover and diversity of biocrust-forming mosses and lichens, and increases the physiological stress of soil microbial communities in a semi-arid Pinus halepensis plantation
Source: Front Microbiol. 2015 Aug 25;6:865. doi: 10.3389/fmicb.2015.00865 (PMC4548238; doi:10.3389/fmicb.2015.00865)
Supplement: Supplementary file 1 [file Presentation_1.PDF]

Supplementary Material

Warming reduces biocrust cover and diversity and increases the physiological stress of soil microbial communities in a semi-arid *Pinus halepensis* plantation

Fernando T. Maestre[1,ρ,*], Cristina Escolar[1,ρ], Richard D. Bardgett[2], Jennifer A. J. Dungait[3], Beatriz Gozalo[1] & Victoria Ochoa[1]

[1]Área de Biodiversidad y Conservación, Departamento de Biología y Geología, Física y Química Inorgánica, Escuela Superior de Ciencias Experimentales y Tecnología, Universidad Rey Juan Carlos, E-28933 Móstoles, Spain
[2]Faculty of Life Sciences, Michael Smith Building, The University of Manchester, Manchester, M13 9PT, UK
Sustainable Soils and Grassland Systems Department, Rothamsted Research, North Wyke, Okehampton, Devon EX20 2SB, UK
[ρ]Both authors equally contributed to this work
[*]Corresponding author: Phone: +34914888115; Fax: +34916647490; E-mail fernando.maestre@urjc.es

A)

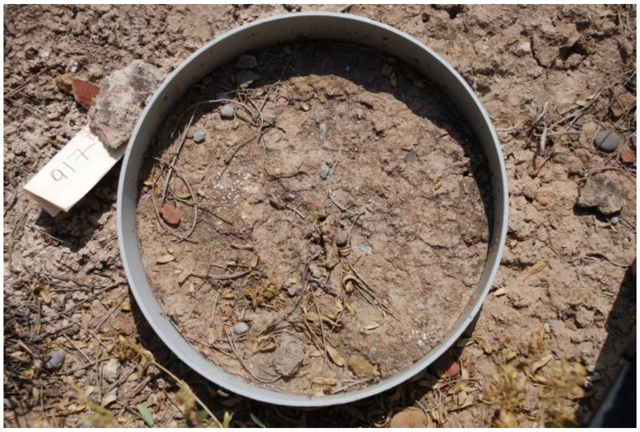

B)

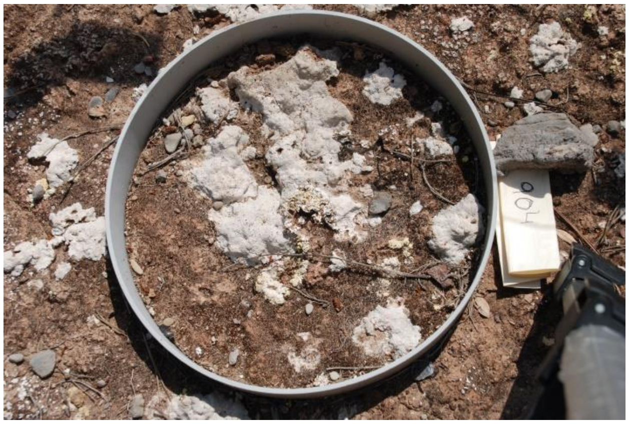

C)

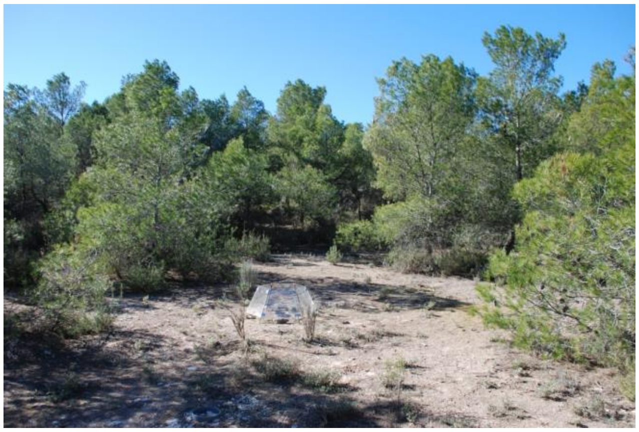

D)

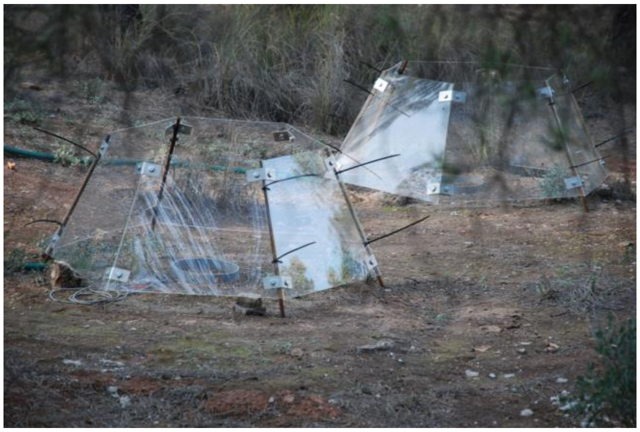

Figure S1. View of a low (A) and a high (B) biocrust cover plot. Partial view of the study area (C) and detailed view of an experimental plot with an open top chamber (D).

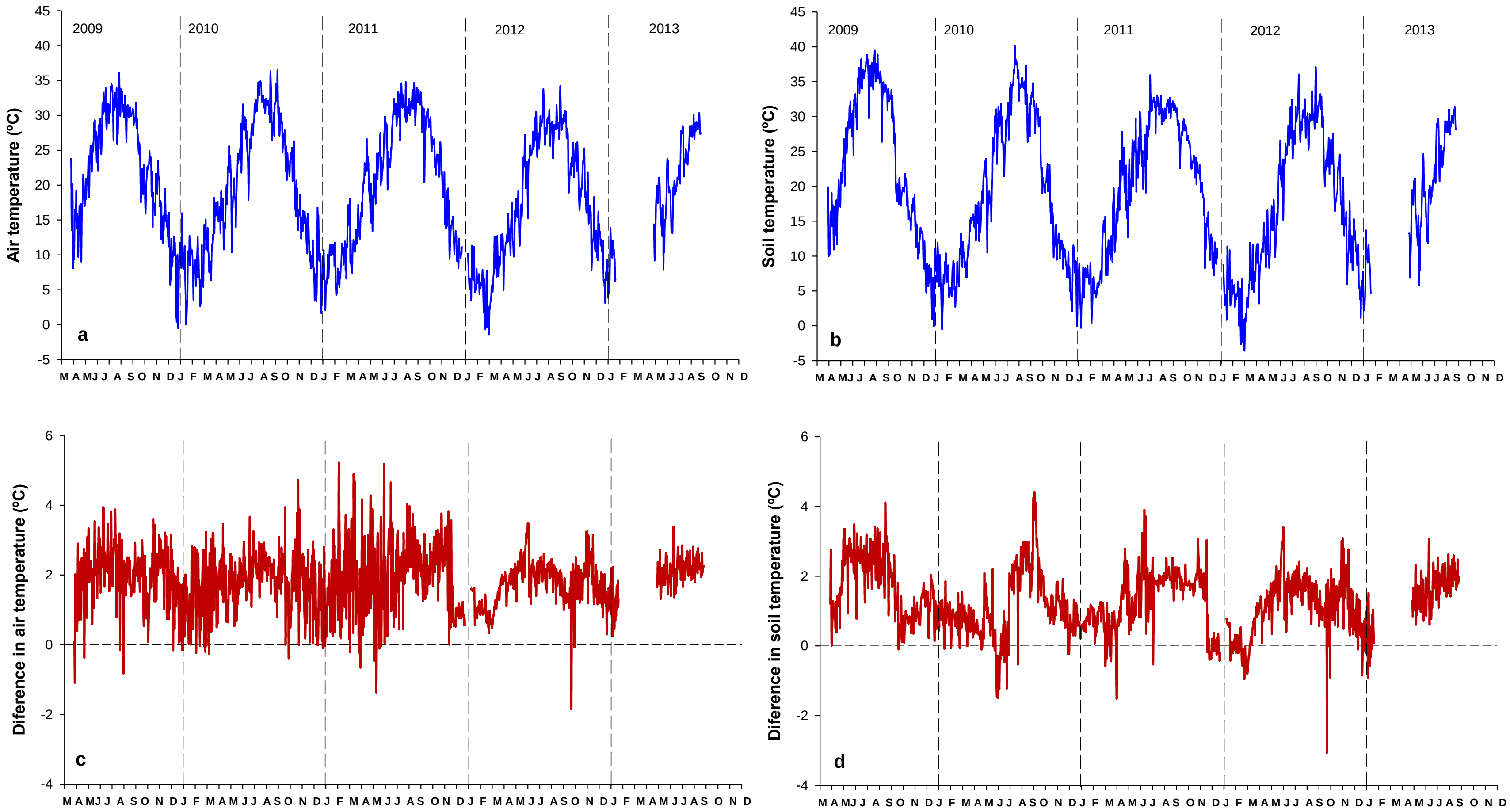

Figure S2. Air (a) and soil (b) temperature in the control treatment throughout the duration of the experiment, and effects of the warming treatment on this variable (differences with the temperature registered in the control treatment; c, d). Data represent daily means.

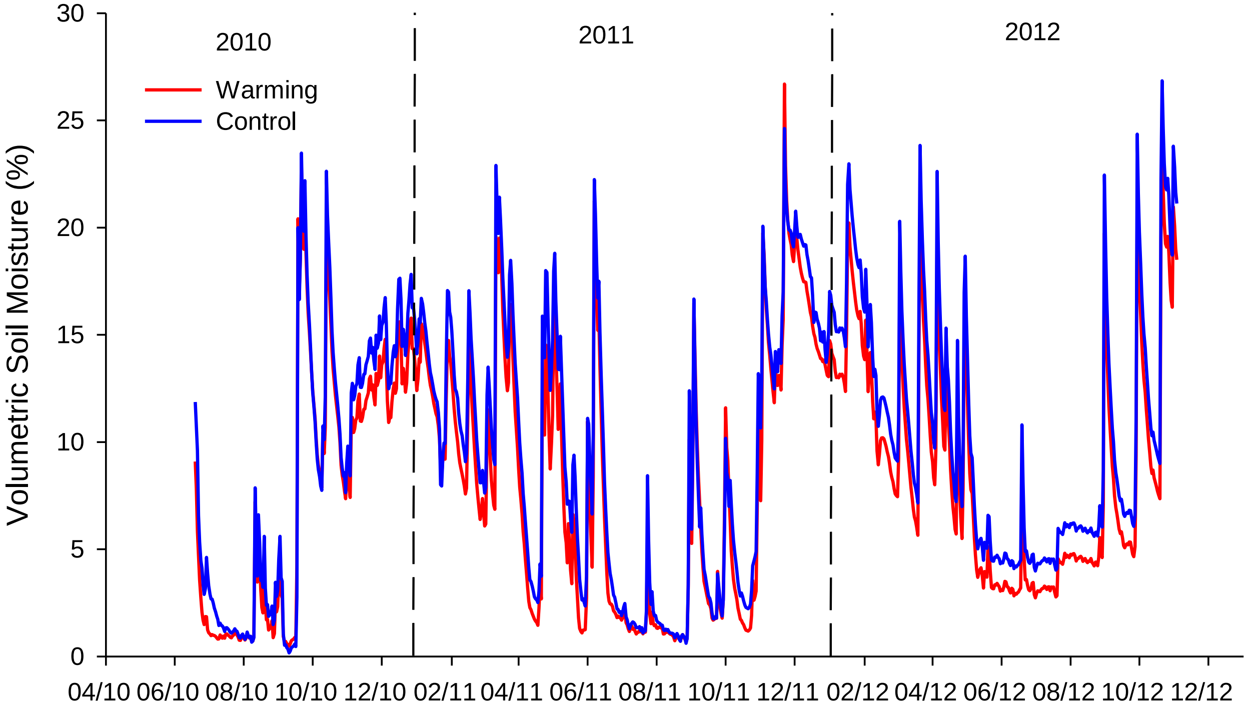

Figure S3. Soil moisture (0-5 cm depth) in control and warming treatments throughout the duration of the experiment. Due to malfunction of the sensors, no data are available beyond December 2012.

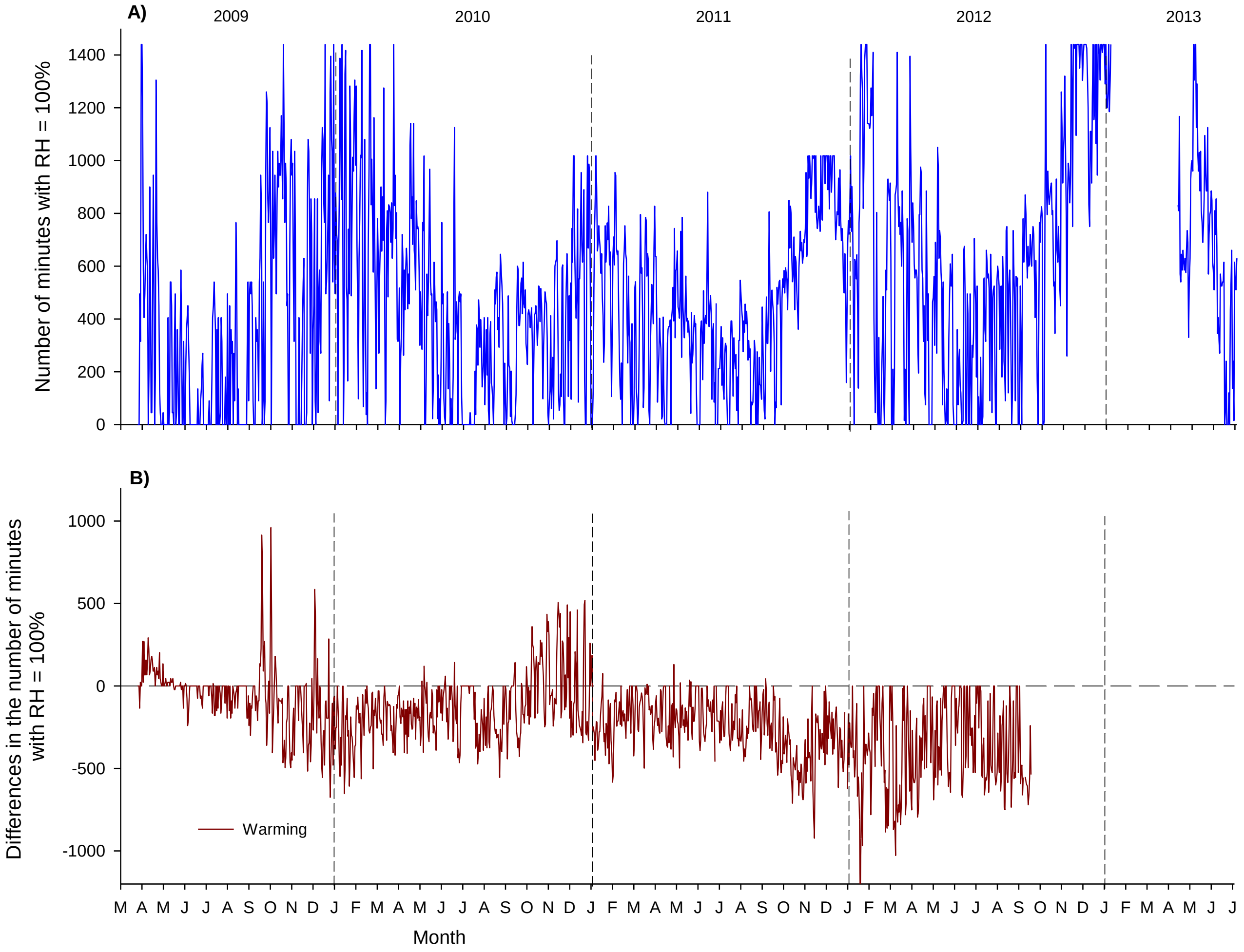

Figure S4. Number of minutes/day when air relative humidity (RH) was 100% in the control treatment (A), and effects of warming on this variable (measured as the difference with respect to the control treatment, B) throughout the duration of the experiment. Negative values in the panel B indicate that the warming treatment is reducing the duration of those periods with RH = 100%. Due to malfunction of the sensors, no data were available in the warming treatment beyond October 2012.

Table S1. Checklist and frequency of lichens and bryophytes in the different treatments at the beginning of the experiment (February 2009) and 53 months later (June 2013). WA = warming treatment.

**February 2009**	**Low biocrust cover**		**High biocrust cover**	
**Species**	**Control**	**WA**	**Control**	**WA**
Fulgensia subbracteata (Nyl.) Poelt.	100	100	100	100
Diploschistes diacapsis (Ach.) Lumbsch.	70	50	100	90
Tortula revolvens (Schimp.) G. Roth var. *obtusata* Reimers	70	60	80	100
Toninia sedifolia (Scop.) Timdal.	40	10	80	70
Collema crispum (Huds.) F.H. Wigg.	40	20	60	60
Lepraria crassissima (Hue) Lettau	30	10	30	60
Squamarina lentigera (Weber) Poelt.	20	0	20	40
Psora decipiens (Hedw.) Hoffm.	20	20	80	40
Squamarina cartilaginea (With.) P. James.	20	30	80	30
Toninia albilabra (Szatala) Oxner.	10	0	0	0
Acarospora nodulosa (Dufour) Hue.	0	0	20	0
Placidium squamulosum (Ach.) Breuss.	0	10	30	70
**June 2013**	**Low biocrust cover**		**High biocrust cover**	
**Species**	**Control**	**WA**	**Control**	**WA**
Fulgensia subbracteata (Nyl.) Poelt.	100	70	100	60
Tortula revolvens (Schimp.) G. Roth var. *obtusata* Reimers	80	100	90	90
Collema crispum (Huds.) F.H. Wigg.	70	20	60	50
Diploschistes diacapsis (Ach.) Lumbsch.	60	0	90	60
Toninia sedifolia (Scop.) Timdal.	40	0	40	40
Squamarina lentigera (Weber) Poelt.	40	20	80	20
Psora decipiens (Hedw.) Hoffm.	20	10	50	40
Squamarina cartilaginea (With.) P. James.	10	0	30	20
Lepraria crassissima (Hue) Lettau	0	10	0	50
Toninia albilabra (Szatala) Oxner.	0	0	0	0
Acarospora nodulosa (Dufour) Hue.	0	0	10	0
Placidium squamulosum (Ach.) Breuss.	0	0	0	0

Table S2. PERMANOVA results for main treatment effects and interactions on the whole PLFA matrix data at the beginning of the experiment and 16 and 53 months after. BSC = biocrust cover (low vs. high cover plots), WA = warming (control vs. 2ºC warming).

Initial Time	Source	df	SS	MS	Pseudo-F	*P*(perm)
	BSC	1	847.40	847.40	1.11	0.309
	WA	1	1484.30	1484.30	1.95	0.133
	BSC x WA	1	1631.40	1631.40	2.15	0.109
	Res	36	27372	760.33		
	Total	39	31335			
16 Months Later						
	BSC	1	791.73	791.73	1.06	0.339
	WA	1	627.70	627.70	0.84	0.451
	BSC x WA	1	604.26	604.26	0.81	0.435
	Res	16	11927	745.43		
	Total	19	13951			
53 months Later						
	BSC	1	56.99	56.99	0.16	0.895
	WA	1	125.95	125.95	0.35	0.669
	BSC x WA	1	86.60	86.60	0.24	0.800
	Res	16	5727.70	357.98		
	Total	19	5997.20			

Table S3. ANOVA results for main treatment effects and interactions on major microbial groups and the cy17:0/16:1ω7 ratio at the beginning of the experiment and 16 and 53 months after. BSC = biocrust cover (low vs. high cover plots), and WA = warming. P values below 0.05 are in bold.

Gram (+)

Initial Time	Source	df	SS	MS	F	P
	BSC	1	0.60	0.60	0.08	0.783
	WA	1	4.30	4.30	0.56	0.461
	BSC x WA	1	2.67	2.67	0.35	0.560
	Res	36	278.67	7.74		
	Total	39	286.24			
16 Months Later	Source	df	SS	MS	F	P
	BSC	1	26.38	26.38	1.52	0.235
	WA	1	18.45	18.45	1.07	0.317
	BSC x WA	1	36.86	36.86	2.13	0.164
	Res	16	276.88	17.30		
	Total	19	358.56			
53 months Later	Source	df	SS	MS	F	P
	BSC	1	13.68	13.68	0.51	0.486
	WA	1	37.48	37.48	1.39	0.255
	BSC x WA	1	9.41	9.41	0.35	0.563
	Res	16	430.83	26.93		
	Total	19	491.41			

Gram (-)

Initial Time	Source	df	SS	MS	F	*P*
	BSC	1	0.95	0.95	0.42	0.520
	WA	1	0.03	0.03	0.01	0.911
	BSC x WA	1	0.03	0.03	0.01	0.916
	Res	36	81.10	2.25		
	Total	39	82.10			
16 Months Later	Source	df	SS	MS	F	*P*
	BSC	1	4.74	4.74	1.53	0.234
	WA	1	1.44	1.44	0.46	0.505
	BSC x WA	1	4.12	4.12	1.33	0.265
	Res	16	49.53	3.10		
	Total	19	59.83			
53 months Later	Source	df	SS	MS	F	*P*
	BSC	1	0.35	0.35	0.17	0.685
	WA	1	1.34	1.34	0.66	0.430
	BSC x WA	1	1.49	1.49	0.73	0.406
	Res	16	32.76	2.05		
	Total	19	35.94			

Fungi

Initial Time	Source	df	SS	MS	F	*P*
	BSC	1	0.27	0.27	0.49	0.487
	WA	1	0.26	0.26	0.48	0.494
	BSC x WA	1	0.97	0.97	1.76	0.193
	Res	36	19.83	0.55		
	Total	39	21.33			
16 Months Later	Source	df	SS	MS	F	*P*
	BSC	1	4.85	4.85	1.11	0.308
	WA	1	0.09	0.09	0.02	0.890
	BSC x WA	1	0.78	0.78	0.18	0.679
	Res	16	70.04	4.38		
	Total	19	75.76			
53 months Later	Source	df	SS	MS	F	*P*
	BSC	1	0.28	0.28	0.06	0.817
	WA	1	0.06	0.06	0.01	0.913
	BSC x WA	1	1.52	1.52	0.30	0.590
	Res	16	80.18	5.01		
	Total	19	82.03			

Actinobacteria

Initial Time	Source	gl	SS	MS	F	*P*
	BSC	1	0.00	0.00	0.01	0.921
	WA	1	0.34	0.34	0.68	0.413
	BSC x WA	1	0.24	0.24	0.49	0.490
	Res	36	18.08	0.50		
	Total	39	18.68			
16 Months Later	Source	df	SS	MS	F	*P*
	BSC	1	0.15	0.15	0.24	0.632
	WA	1	0.00	0.00	0.00	0.996
	BSC x WA	1	0.08	0.08	0.12	0.735
	Res	16	10.38	0.65		
	Total	19	10.62			
53 months Later	Source	df	SS	MS	F	*P*
	BSC	1	2.88	2.88	1.50	0.238
	WA	1	1.41	1.41	0.74	0.404
	BSC x WA	1	0.77	0.77	0.40	0.535
	Res	16	30.67	1.92		
	Total	19	35.74			

Total bacteria

Initial Time	Source	gl	SS	MS	F	*P*
	BSC	1	3.10	3.10	0.18	0.671

	Source	df	SS	MS	F	*P*
	WA	1	2.71	2.71	0.16	0.691
	BSC x WA	1	1.51	1.51	0.09	0.767
	Res	36	609.24	16.92		
	Total	39	616.55			
16 Months Later	Source	df	SS	MS	F	*P*
	BSC	1	49.64	49.64	1.57	0.229
	WA	1	26.15	26.15	0.83	0.377
	BSC x WA	1	60.66	60.66	1.91	0.186
	Res	16	507.02	31.69		
	Total	19	643.47			
53 months Later	Source	df	SS	MS	F	*P*
	BSC	1	17.28	17.28	0.44	0.516
	WA	1	41.33	41.33	1.05	0.320
	BSC x WA	1	17.02	17.02	0.43	0.520
	Res	16	628.02	39.25		
	Total	19	703.65			

Fungi:bacteria

Initial Time	Source	gl	SS	MS	F	*P*
	BSC	1	0.01	0.01	0.84	0.366
	WA	1	0.03	0.03	2.34	0.135
	BSC x WA	1	0.00	0.00	0.18	0.673
	Res	36	0.47	0.01		
	Total	39	0.51			
16 Months Later	Source	df	SS	MS	F	*P*
	BSC	1	0.00	0.00	0.00	0.949
	WA	1	0.02	0.02	0.97	0.340
	BSC x WA	1	0.04	0.04	1.90	0.187
	Res	16	0.30	0.02		
	Total	19	0.35			
53 months Later	Source	df	SS	MS	F	*P*
	BSC	1	0.01	0.01	0.53	0.478
	WA	1	0.03	0.03	1.07	0.316
	BSC x WA	1	0.00	0.00	0.08	0.783
	Res	16	0.41	0.03		
	Total	19	0.45			

cy17:0/16:1ω7 ratio

Initial Time	Source	gl	SS	MS	F	*P*
	BSC	1	0.04	0.04	0.64	0.429
	WA	1	0.21	0.21	3.45	0.071
	BSC x WA	1	0.08	0.08	1.41	0.244
	Res	36	2.14	0.06		

	Total	39	2.47			
16 Months Later	Source	df	SS	MS	F	*P*
	BSC	1	0.00	0.00	0.06	0.814
	WA	1	0.19	0.19	5.11	**0.038**
	BSC x WA	1	0.05	0.05	1.50	0.238
	Res	16	0.58	0.04		
	Total	19	0.83			
53 months Later	Source	df	SS	MS	F	*P*
	BSC	1	0.43	0.43	2.96	0.105
	WA	1	1.13	1.13	7.73	**0.013**
	BSC x WA	1	0.17	0.17	1.15	0.300
	Res	16	2.33	0.15		
	Total	19	4.06			
**Cyanobacteria**						
Initial Time	Source	gl	SS	MS	F	*P*
	BSC	1	0.00	0.70	0.76	0.389
	WA	1	0.04	0.00	0.00	0.999
	BSC x WA	1	32.97	0.04	0.04	0.843
	Res	36	59.53	0.92		
	Total	39	33.70			
16 Months Later	Source	df	SS	MS	F	*P*
	BSC	1	1.88	1.88	1.59	0.225
	WA	1	1.31	1.31	1.11	0.308
	BSC x WA	1	1.87	1.87	1.58	0.227
	Res	16	18.95	1.18		
	Total	19	24.02			
53 months Later	Source	df	SS	MS	F	*P*
	BSC	1	0.02	0.02	0.03	0.864
	WA	1	1.51	1.51	1.86	0.192
	BSC x WA	1	0.59	0.59	0.72	0.408
	Res	16	12.99	0.81		
	Total	19	15.11			

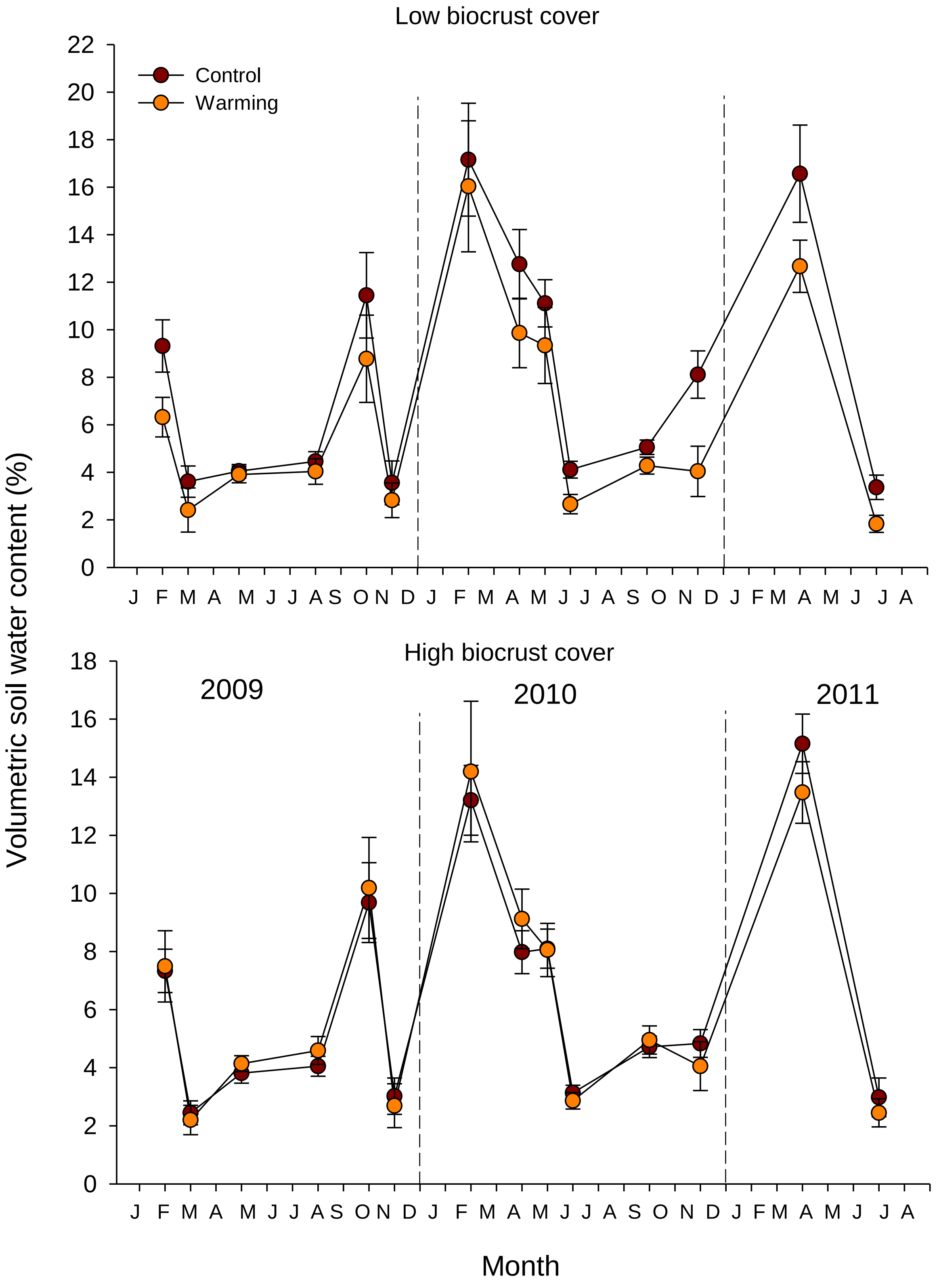

Figure S5. Volumetric soil moisture (0-5 cm depth) measured between January 2009 and August 2011 in the low and high biocrust cover plots. Data represent means + SE (n = 10).
